# Subcutaneous Maturation of Neural Stem Cell-Loaded Hydrogels Forms Region-Specific Neuroepithelium

**DOI:** 10.3390/cells7100173

**Published:** 2018-10-17

**Authors:** Mahmoud Farrag, Nic D. Leipzig

**Affiliations:** 1 Integrated Bioscience Program, The University of Akron, Akron, OH 44325, USA; mfa14@zips.uakron.edu; 2 Department of Chemical and Biomolecular Engineering, The University of Akron, Akron, OH 44325, USA

**Keywords:** chitosan-based scaffold, NSCs, adult-derived neural stem cells, stem cell priming, preconditioning of neural stem cells, region-specific neural tissue, neural identity, instructive biomaterials

## Abstract

A combinatorial approach integrating stem cells and capable of exploiting available cues is likely needed to regenerate lost neural tissues and ultimately restore neurologic functions. This study investigates the effects of the subcutaneous maturation of adult-derived neural stem cell (aNSCs) seeded into biomaterial constructs on aNSC differentiation and ultimate regional neuronal identity as a first step toward a future spinal cord injury treatment. To achieve this, we encapsulated rat aNSCs in chitosan-based hydrogels functionalized with immobilized azide-tagged interferon-γ inside a chitosan conduit. Then, we implanted these constructs in the subcutaneous tissues in the backs of rats in the cervical, thoracic, and lumbar regions for 4, 6, and 8 weeks. After harvesting the scaffolds, we analyzed cell differentiation qualitatively using immunohistochemical analysis and quantitatively using RT-qPCR. Results revealed that the hydrogels supported aNSC survival and differentiation up to 4 weeks in the subcutaneous environment as marked by the expression of several neurogenesis markers. Most interesting, the aNSCs expressed region-specific Hox genes corresponding to their region of implantation. This study lays the groundwork for further translational work to recapitulate the potentially undiscovered patterning cues in the subcutaneous tissue and provide support for the conceptual premise that our bioengineering approach can form caudalized region-specific neuroepithelium.

## 1. Introduction

Spinal cord injury (SCI) is typically a life changing event as it leads to loss of neural tissues and their associated motor, sensory, and autonomic functions at and below the level of injury. Data from several studies [1,2,3] suggest that cell-based therapies present a promising solution for treatment of SCI and can reproduce, at least partially, the development process; however, there is little agreement on what are the best strategies to implement. Specifically, aNSCs represent an important potential candidate for cell-based therapies for many reasons as spinal cord development begins with NSCs [4]. Also, they have been proven to differentiate into CNS-specific neurons, astrocytes and oligodendrocytes [4,5,6,7,8,9,10]. Moreover, their role extends beyond reconstructing lost tissues. They contribute to their surrounding extracellular environment through the production of cytokines [11,12,13] and neurotrophic factors [14,15], in addition to their known immunomodulation role [16,17,18]. aNSCs are also outside many of the ethical controversies surrounding the derivation and use of embryonic stem cells. Moreover, aNSCs can be expanded in culture using well-documented mitogens. Although the advantages of utilizing aNSCs are many, it is not devoid of technical hurdles. The small number of cells obtained from human brain samples and availability of human samples are major limitations. These limitations have been addressed through technical and scientific approaches that maximize the yield of these samples of aNSCs and through cryobanking [19,20].

Previous research has established that NSCs can cross the injury chasm by growing axons that integrate with host neural tissues [1]. However, this integration is inadequate because even with exposure to known developmental patterning cues, aNSCs are not able to recapitulate the developmental signaling pathways or demonstrate region-specific identity [21,22], thus directly limiting their integration. Recent developments have led to a growing interest in preconditioning transplanted cells to withstand the hostility of the injury site and improve their integration. Several attempts have been made to precondition cells in general, and NSCs in particular. Approaches have included exposure to hypoxia and other harsh conditions, cytokines or small molecules before implantation to improve engraftment and have proved to be effective over cells that were not preconditioned [23,24,25,26,27,28,29]. Moreover, a growing body of research recognizes the critical role played by the extracellular molecular environment in determining survival and neurogenic capacity of aNSCs when they are transplanted [30,31,32]. Finally, the subcutaneous adipose tissue is known to be unique in its molecular signatures [33]; however, to date there has been no detailed investigation of the effect of subcutaneous environment and its molecular cues on aNSCs differentiation for preconditioning. These cues may be able to stimulate the spatial organization of neurons within the spinal cord and guide aNSCs caudalization to acquire spinal cord region-specific identity.

The Hox code (the pattern of Hox genes expression that is specific for each body region [34]) drives the spatial organization of spinal cord motor neurons along the craniocaudal axis. This gene family of transcription factors comprises 39 highly conserved factors that are categorized into 4 paralogous groups; A, B, C, and D. Within each group, the members are numbered from 1 to 13 (reviewed in [34,35,36,37,38,39]). Existing research recognizes the critical role played by Hox genes in stem cell differentiation and fate decisions [40,41]. Because Hox gene expression is so tightly regulated/conserved it denotes the attainment of regional identity of developing structures, particularly the neuroepithelium, along the craniocaudal axis [35,37,42,43]. Therefore, the success of any approach guiding the regional identity of stem cells and their progeny should include the evaluation of Hox expression.

Bioengineering strategies capable of simultaneously providing differentiation signals and responding to exogenous signals offer tremendous promise for regenerative medicine (more comprehensive reviews can be found in [44,45,46,47,48,49]). Further, biomaterial conduits can represent a physical barrier between encapsulated cells and host immune response [50] until these cells can adapt to their new environment and respond to the existing chemical and physical cues favorably. Previous studies from our group have demonstrated that chitosan, a structural mimetic of hyaluronic acid (HA), a major extracellular protein in CNS, induces minimal immune response when implanted in vivo [7] and can be used as a platform for biomaterial constructs to direct NSCs differentiation both in vitro [6] and in vivo [7,8]. Among signaling proteins available to induce aNSCs differentiation, immobilized interferon-γ (IFN-γ) encourages one of the most robust differentiation responses available via direct cellular signaling mechanisms [51] and immobilization of the active sequence via a recombinant fusion protein is a key to containing signaling within the conduit to circumvent immune cell recruitment [5,7,8,9,10]. Moreover, pervasive in the field of neurotrauma is the fact that IFN-γ is thought to only act as a pro-inflammatory cytokine with negative consequences, and therefore its use in CNS injuries is controversial. Several studies recently have challenged this concern, at least in respect to SCI, and showed benefits to IFN-γ signaling with better functional outcomes in SCI models (reviewed in [52]).

This study aims to lay the groundwork for a new paradigm in SCI treatment using a stem cell-based therapeutic approach that preconditions aNSCs subcutaneously, which is more likely to improve engraftment. Therefore, this study primarily attempts to prove that a subcutaneous implantation approach can guide the differentiation of aNSCs to acquire region-specific neuronal identity based on the region of implantation before use as a functional graft. Our main hypothesis was that implantation of aNSC-MAC constructs in the subcutaneous environment will help newly differentiated neurons acquire region-specific identity according to the region of implantation, while maintaining stem cell potency. The importance and originality of this study are that it explores the role of the subcutaneous environment in preconditioning and steering aNSCs toward neuroepithelium with craniocaudal regional identity resembling the spinal cord motor regional identity. Generating neuroepithelium with predictable region-specific identity is a leap forward for regenerative medicine. In this study, aNSC encapsulated neurogenic conduits were synthesized and implanted in the subcutaneous environment of the midline of the back of rats for 4, 6, and 8 weeks. A combination of quantitative and qualitative approaches was used in the analysis of different markers to verify aNSC responses to achieve our overall objective.

## 2. Materials and Methods

### 2.1. Methacrylamide Chitosan (MAC) Preparation

MAC was prepared by adapting the procedure used by Li et al. [8]. To begin the process, 3 wt % *w/v* chitosan (Protosan UP B80/20, NovaMatrix, Drammen, Norway) was dissolved and methacrylic anhydride (Sigma-Aldrich, St. Louis, MO, USA) was then added at 0.4 molar ratio. The mixture was allowed to stir for 3+ h. Following stirring, the mixture was dialyzed (12–14 kDa dialysis tubing, Spectrum Labs, Waltham, MA, USA) against distilled water and then freeze-dried. Finally, the product was stored in −20 °C until use.

### 2.2. MAC-DBCO Preparation for Azide-Tagged IFN-γ (azIFN-γ) Immobilization

Recently, we have developed a simple bio-orthogonal method of protein immobilization in our lab to immobilize IFN-γ to MAC. More details about this method, as well as about design, expression and purification of recombinant N-terminal azide-tagged interferon-γ, can be found in our previous study [10]. Briefly, 2 wt % MAC in PBS was prepared and Dibenzocyclooctyne-*N*-hydroxysuccinimidyl (DBCO-NHS) ester was added and allowed to react by shaking at room temperature (RT) for 3 h. The material was dialyzed against deionized (DI) water (12–14 kDA dialysis tube, Spectrum Labs) and then lyophilized. Finally, the product was stored in −20 °C until use.

### 2.3. Chitosan Conduit Synthesis

The synthesis of chitosan tubes was performed according to procedures described before [52,53]. Briefly, 3 wt % chitosan and EtOH were mixed at a ratio of 1:1. After degassing the chitosan solution, 89 μL acetic anhydride was added per each 5 mL of chitosan solution and the mixture was injected into a mold formed of a suspended steel rod (OD: 4.3 mm) (McMaster Carr, Aurora, OH, USA) in a glass tube. A chitin tube was formed in the mold overnight. The chitin tube was deacetylated by hydrolysis in 40% NaOH at 100 °C for 4 h to change into chitosan tube. Following deacetylation, the tube was cut into 10 mm long conduits and allowed to air-dry overnight. Finally, the conduits were rehydrated then stored until used. Prior to use, conduits were sterilized by immersing in 70% EtOH for at least one hour.

### 2.4. Photoreactive Laminin

To enable laminin to covalently attach to the MAC material during UV crosslinking and to enhance NSC adhesion laminin (Life Technologies, Carlsbad, CA, USA) was mixed with acrylic acid *N*-hydroxysuccinimide ester (Sigma-Aldrich, St. Louis, MO, USA) and reacted for 4 h at 4 °C. The solution was then purified by dialysis against 1× PBS with multiple buffer changes for three days. The concentration was then measured using a spectrophotometer (Infinite M200 with NanoQuant Plate, Tecan, Grödig, Austria).

### 2.5. aNSC Harvest and Culture

All procedures involving animals were approved by the University of Akron institutional animal care and use committee (IACUC, protocol #15-02-3-LRD). aNSCs were harvested from the SVZ of the lateral ventricles using the same method that was detailed previously [6,8]. Adult female Fischer 344 rats (6–8 weeks old, Envigo, Haslett, MI, USA) were euthanized using CO_2_. Harvested tissues were dissociated chemically using a papain dissociation kit (Worthington Biochemical Corporation, Lakewood, NJ, USA) according to the company’s instructions. aNSCs were expanded as neurospheres in chemically defined serum-free growth medium. Cells were counted and passaged every week and low passage number (3–5) cells were used for encapsulation into the hydrogel.

### 2.6. NSC Encapsulation in MAC Hydrogels

The first step in assembly of the neurogenic scaffolds was to dissolve lyophilized MAC-DBCO in ultrapure water at a concentration of 3%. After dissolving, the solution was autoclaved. The second step was to react azIFN-γ (300 ng/mL) with MAC-DBCO by stirring at 4 °C overnight. In the meantime, a stock of photo-initiator, which is 1-hydroxycyclohexyl phenyl ketone (IRG-184; Sigma-Aldrich, St. Louis, MO, USA) at 300 mg/mL in 1-vinyl-2-pyrrolidinone (Sigma-Aldrich, St. Louis, MO, USA), was prepared. To the azIFN-γ /MAC-DBCO solution, we added 50 mg/g photo-reactive laminin, 3 million cells/gram material, 14.7 mM IRG-184. To attain 2 wt % buffered MAC-DBCO solution, we lastly add 10× PBS to reach the final volume. When all components were added, this resulting mixture was gently mixed (0.5–1 min, 1500 RPM, SpeedMixer DAC 150 FVZ, Hauschild Engineering, Hamm, Germany) and then 150–200 µL of the mixture was transferred to a chitosan conduit. Finally, free radical polymerization was achieved by exposure to UV (365 nm, 2.7 mW/cm^2^) light for about 3 min.

### 2.7. Implantation of the aNSC-MAC System Subcutaneously

**Figure 1:**
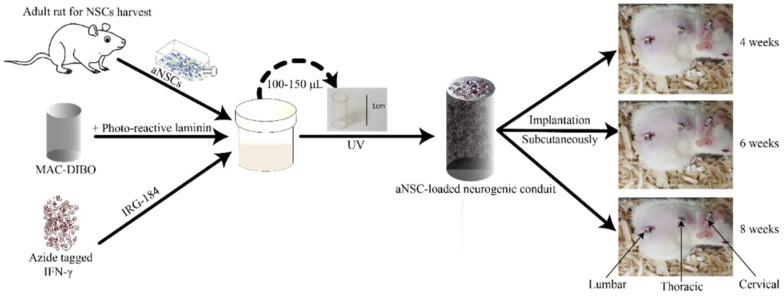
Schematic of overall study design. aNSCs are mixed with IFN-γ bio-orthogonal functionalized MAC-DIBO in the presence of photo-reactive laminin (via acrylated lysine residues) and photoinitiator (IRG-184), transferred to the inside of a preformed chitosan conduit, then exposed to UV light to form a hydrogel. These hydrogels were then implanted in the subcutaneous tissue of the back of adult rats, along the midline, in regions corresponding to the cervical, thoracic and lumbar segments of the spinal cord to test the effects of regional maturation. They were left for 4, 6, and 8 weeks before being harvested and analyzed using gene expression and IHC.

Subcutaneous implantation of the cell-biomaterial scaffolds was performed under anesthesia and complete aseptic conditions using adult female Fischer 344 rats (8 weeks old, Envigo, Haslett, MI, USA). First, we created 3 separate midline incisions, approximately 5–10 mm in length. The most caudal lies about 30 mm from the base of the tail over the lumbar vertebrae. The other two incisions lie along the thoracic and cervical vertebrae (Figure 1). Following incisions, a subcutaneous space deep to each incision was created by blunt dissection. A one cell-biomaterial scaffold was inserted parallel to the midline in the created pouch. Finally, the incisions were closed using Michel clips. The scaffolds were allowed to mature in the subcutaneous environment for 4, 6, and 8 weeks. At each time point, the animals were euthanized by CO_2_, and scaffolds were harvested. Then, each conduit was cut into halves: one half, randomly selected, was used for qualitative immunohistological analysis and the other half was used for quantitative analysis using qPCR.

### 2.8. Immunohistochemistry (IHC)

Immediately after the harvest of the scaffolds, the halves assigned for IHC were gently washed in PBS and then post-fixed in 4% PFA in PBS. Prior to paraffin embedding, we processed the samples using an automated tissue processor (Model ASP 300S; Leica, Wetzlar, Germany) according to an established protocol. Following processing, the samples were embedded in wax and then left to cool before moving to −20 °C until sectioning. We collected 12 µm cross-sections using a microtome (Model RM2255; Leica, Wetzlar, Germany).

To start IHC, sections on slides were heated in 60 °C oven and then deparaffinized using xylene. The selection of these sections considered nearby sections for staining with different neurogenesis and neuronal markers. The slides were then rehydrated. After deparaffinization, we permeabilized the sections with 0.1% Triton X-100 (Sigma-Aldrich, St. Louis, MO, USA). Next, we blocked with 10% suitable serum in 1× PBS at RT and then washed. The sections were then incubated with the respective primary antibody overnight at 4 °C. We stained selected sections with monoclonal mouse anti-β-III tubulin (1:500, Covance, Princeton, NJ, USA), rabbit monoclonal anti-neuronal nuclei antigen (NeuN) (1:500, Abcam, Cambridge, MA, USA), and rabbit polyclonal anti-FezF2 (1:500, Millipore, Millerica, MA, USA) to identify neurons, monoclonal mouse anti-nestin (1:10, DSHB) to stain undifferentiated neural stem cells, rabbit polyclonal anti-paired box protein-6 (Pax6) (1:100, Abcam, Cambridge, MA, USA) to assay for the presence of embryonic neuroectoderm differentiation transcription factors, and in addition to rabbit polyclonal anti-Sox1 (1:500, Abcam, Cambridge, MA, USA), which is a CNS-specific marker and it marks the induction of neuroectoderm. Prior to incubating sections with secondary antibodies, sections were washed three times with PBS, with each wash lasted at least 15 min. The incubation lasted for at least one hour and was followed by an additional three 15 min PBS washes. The final stage was staining cell nuclei with 10 mM Hoechst 33342 for 7 min followed by mounting using ProLong™ Gold Antifade (ThermoFisher, Waltham, MA, USA). We used an Olympus IX81 fluorescent microscope or an Olympus FV1000 confocal microscope to image slides, then we processed the images using MetaMorph software version 7.10 (Molecular Devices, Sunnyvale, CA, USA).

### 2.9. RT-qPCR

The scaffold segments assigned to molecular analysis were immediately stored in RNA*later* in 4 °C overnight before moving it to −80 °C until further processing. Also, NSCs from the same batch were stored to be used as *t* = 0 controls for Hox gene expression studies. Before extracting RNA from the neurogenic scaffolds, we removed the chitosan tubes to reduce the polysaccharide load that can entrap RNA during extraction. We extracted RNA using RNeasy extraction mini kit (Qiagen, Valencia, CA, USA) following the company’s protocol. Following RNA extraction, we measured RNA concentration and purity using spectrophotometer (Infinite M200 with NanoQuant Plate, Tecan, Grödig, Austria) or Nanodrop spectrophotometer (Thermo Scientific, Wilmington, DE, USA). We excluded RNA samples with concentration lower than 10 ng/µL or 260/280 ratio less than 1.8 from further processing. Extracted RNA was stored in −80 °C until used to make cDNA. Prior to cDNA synthesis, we equilibrated all sample concentrations to 20 ng/µL. The synthesis of cDNA was performed using the Transcriptor First Strand cDNA Synthesis Kit (Roche, Mannheim, Germany) according to the company’s protocol.

#### 2.9.1. Absolute Quantification Analysis of Target Genes

In an attempt to make the comparison between gene expression in each region more representative numerically, absolute quantitative analysis for gene expression was performed. This analysis requires a gene product with a known number of copies to build a standard curve. To begin the process, we ran conventional PCR on each target gene using FastStart Taq DNA polymerase (Roche, Mannheim, Germany) according to the company’s instructions. Next, we ran 2% agarose gel electrophoresis (UltrapureTM Agarose 1000, Invitrogen, Carlsbad, CA, USA) with subsequent ethidium bromide staining to evaluate the PCR results before cloning. Once we obtained positive results for our target genes, we cloned our genes using a TOPO TA Cloning Kit (Invitrogen, Carlsbad, CA, USA) according to the company’s protocol. Next, we isolated plasmid preparations using E.Z.N.A.^®^ Plasmid Mini Kit II (Omega bio-tek, Norcross, GA, USA) according to the company’s protocol and measured plasmid concentrations using a Nanodrop spectrophotometer (Thermo Scientific, Wilmington, DE, USA). The plasmid concentration and number of base pairs were used to determine the number of gene copies of each gene using the URI Genomics and Sequencing Center online calculator (http://cels.uri.edu/gsc/cndna.html). We then could build a standard curve for each gene using serial dilutions of calculated copy number of plasmids. 15 μL qPCR reactions, that included 1 μL DNA template and 250–500 nm of each primer, were run in triplicates on LightCycler 480 (Roche, Mannheim, Germany) using SYBR green detection chemistry. Amplification was achieved with 5 min activation step at 95 °C, followed by 45 cycles of 10 s at 95 °C, 15 s at 60 °C, 10 s at 72 °C with real-time SYBR green dye I fluorescence measurement. The gene primer sets used for these experiments are provided in Supplemental Table S1.

#### 2.9.2. Relative Expression qPCR Assays

To study Hox gene expression in harvested cell-biomaterial scaffolds, we ordered custom-configured TaqMan^®^ Array Plates (cat ID: 4391528, ThermoFisher, Waltham, MA, USA) and TaqMan Fast Advanced Master Mix (Cat #: 4444556, ThermoFisher, Waltham, MA, USA). These plates contained predesigned, configured, and preplated TaqMan gene expression assays that target a cohort of Hox genes in rat species along with endogenous control assays; 18S, GAPDH, and HPRT. Following the company’s protocol and the best-practices, 20 µL reactions were performed using ABI 7300 (Applied Biosystems, Foster City, CA, USA) under the following conditions: initial incubation at 50 °C for 2 min; followed 20 s at 96 °C for enzyme activation then 40 cycles of 95 °C for 3 s and 60 °C for 30 s. Data were processed using DataAssit^TM^ v3.01 software and analyzed using 2^ΔΔ*C*t^ method as described by Livak and Schittgen [54].

### 2.10. Statistics

Statistical analyses were performed using JMP Pro 13 and Prism 7 (GraphPad Software, Inc., San Diego, CA, USA) software. RT-qPCR data were analyzed using ANOVA with Tukey’s post-hoc test with α = 0.05 to detect significant difference between separate groups. Data are shown as mean ± standard error of the mean (SEM) unless otherwise stated.

## 3. Results

### 3.1. Hydrogel Degradation Corresponds to Loss in Gene Expression

**Figure 2:**
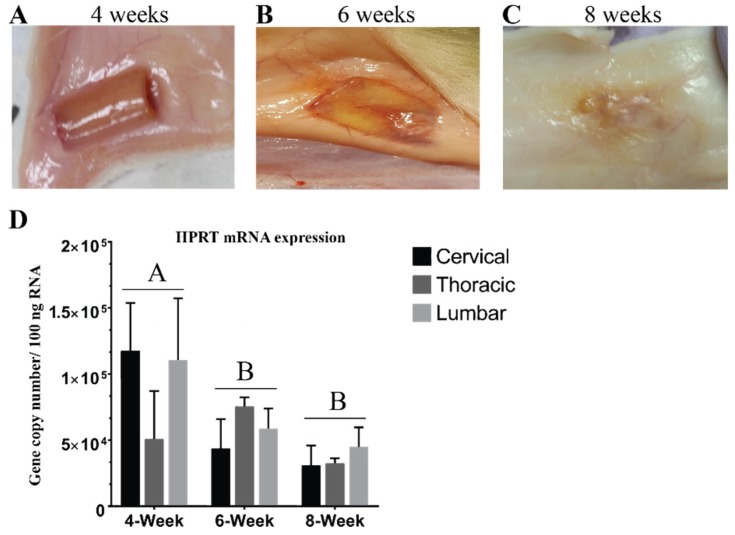
Degradation of subcutaneous conduits began rapidly after 4 weeks subcutaneous maturation. Representative images of the scaffold before harvest at each time point. (**A**) 4-week scaffolds are shown smoothly encapsulated in the subcutaneous tissue with no gross signs of degradation. (**B**) At 6 weeks the scaffolds started to degrade with remnants of the scaffolds still remaining. (**C**) This degradation became more extensive and near-complete at 8 weeks as scaffolds became integrated into the surrounding tissues. (**D**) Shows qPCR absolute quantification data for the reference gene HPRT reported as the number of gene copies in 100 ng total RNA-demonstrating that degradation corresponded with a decreased in expression with longer implantation time. Letters denote significance by two-way ANOVA (*p* < 0.05), mean ± SEM with *n* = 3. There was no statistical difference between different regions.

The first set of analyses examined the impact of the subcutaneous environment on the cell-biomaterial scaffolds over time for assessing potential for future re-transplantation purposes. As such, we implanted aNSC-seeded biomaterial scaffolds in the subcutaneous environment for 4, 6, and 8 weeks then harvested and inspected them for gross changes. At 4 weeks, the scaffolds were found smoothly ensheathed in subcutaneous tissue with no major signs of degradation (Figure 2A). Based on the fact that scaffolds could be removed cleanly, they are likely intact enough that it could be used for a subsequent transplantation such as for SCI treatment. After 4 weeks the neurogenic conduit appeared to degrade rapidly, as indicated by gross signs of degradation at 6 weeks (Figure 2B). Moreover, the degradation became more extensive and near-complete at 8 weeks so that the scaffolds became integrated into the surrounding subcutaneous tissue as shown in Figure 2C. Turning to reference gene expression, Figure 2D demonstrated a decline in gene expression of HPRT with longer implantation time. This trend was also observed in almost all studied genes (Supplemental Figure S1) and corresponds to the observed gross degradation (Figure 2). These observations appeared to be unaffected by implantation region. Based on these results we chose to focus subsequent analyses on the week 4 constructs only.

### 3.2. Stemness Expression Profiles

**Figure 3:**
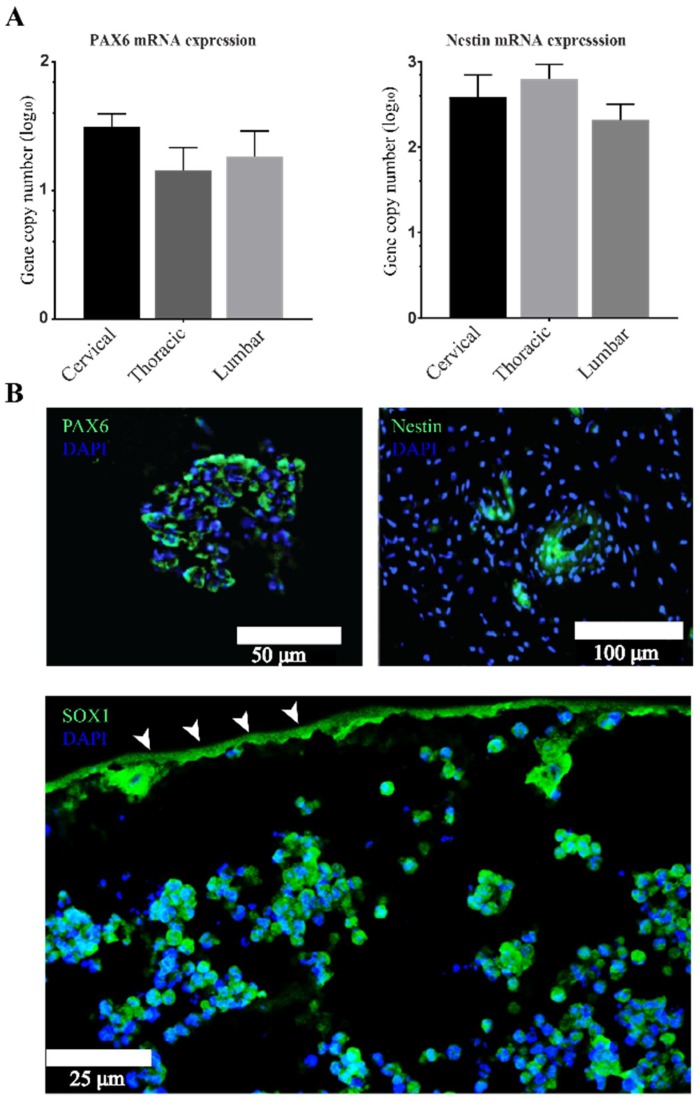
Subcutaneous implantation of the cell-biomaterial system maintains and enhances neural progenitor and neuroepithelium markers expression at 4 weeks. Representative data showing cells within the scaffold expressing the neural progenitor cell marker, nestin, neuroepithelium marker, PAX6, and activated NSC marker, SOX1. (**A**) Shows qPCR absolute quantification data for PAX6 and nestin reported as log_10_ the number of gene copies in 100 ng total RNA. There was no significant difference found between regions in PAX6 or nestin expression by ANOVA (*p* > 0.05) and expressed as mean ± SEM with *n* = 3. (**B**) Shows representative image of IHC staining inside the conduits. Arrowheads point to the auto-fluorescent inner border of the chitosan tube surrounding the biomaterial.

The next set of analyses examined the impact of preconditioning our cell-biomaterial scaffolds in the subcutaneous environment via the ability of aNSCs to maintain their stemness and neuroepithelial markers. Based on our hypothesis, populations of encapsulated aNSCs should be able to maintain stemness marker expression. Moreover, they should express markers representative of various neurogenic developmental stages. To test this hypothesis, IHC and RT-qPCR analyses were employed to assess gene expression of a subset of representative neurogenesis genes at 4 weeks. The next step in analysis was quantitative analysis using absolute RT-qPCR, (Figure 3A), which indicated that conduit encapsulated cells expressed nestin and PAX6. From the IHC data in Figure 3B, it was apparent that aNSCs within the hydrogel expressed protein neuroprogenitor and neuroepithelium markers, nestin and PAX6, respectively. The expression of these markers was observed in sections throughout the hydrogels within the conduits, however, expression was mostly found in aggregations or pockets suggesting maintenance of pools of neural stem/progenitor cells. We also examined Sox1 protein expression using IHC analysis and we found it was expressed similarly to nestin and PAX6 within our system (Figure 3B). These observations demonstrate this biomaterial system supports aNSCs for at least for 4 weeks in the subcutaneous environment, allowing maintenance of subpopulations of stem cells. Interestingly, subcutaneous implantation region did not significantly impact the expression of any of the NSC markers probed. These results show aNSC’s expressed genes that represent several stages of neurogenesis and remarkably demonstrated self-organizing patterns similar to neural rosettes, a hallmark of CNS development (Figure 3B).

### 3.3. Transplanted aNSCs Express Neuronal Markers

**Figure 4:**
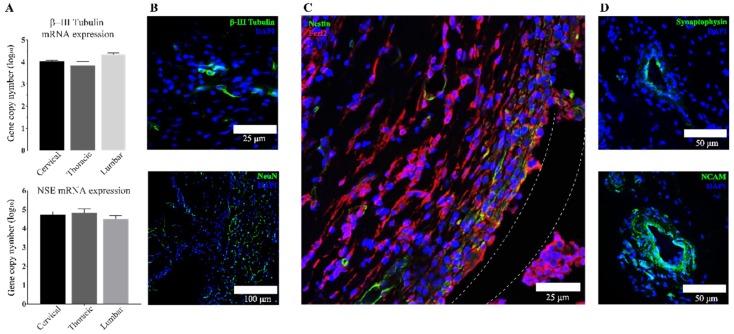
Neuronal marker expression in aNSCs from 4-week scaffolds. (**A**) Shows qPCR absolute quantification data of β-III tubulin and NSE genes reported as log_10_ the number of gene copies in 100 ng total RNA. No significant difference was found in β-III tubulin and NSE expression in different regions by ANOVA (*p* < 0.05). Data reported as mean ± SEM with *n* = 3. (**B**) Shows representative images of IHC staining of β-III tubulin along with the mature neuronal marker, NeuN. (**C**) Shows co-staining of corticospinal tract specific marker, FezF2, along with nestin. Dotted lines represent the edges of the chitosan tube that surrounded the bioscaffold. (**D**) shows representative images of IHC staining of synaptophysin; a neuronal vesicles marker, and NCAM; a binding glycoprotein expressed on the surface of neurons and glial cells.

To further characterize the outcomes of subcutaneous culture and lineage commitment of delivered aNSCs within our system, we investigated the gene expression of the specific neuronal markers β-III tubulin, NSE, and NeuN. β-III tubulin is a widely used neuronal marker as it is a major component of microtubules in neurons. Upregulation of neuron-specific enolase (NSE) expression is most often associated with functional and morphological maturation of neurons, while NeuN marks the initiation of terminal differentiation of neurons and serves as a marker for mature neurons. Figure 4A presents the results obtained from RT-qPCR analysis of β-III tubulin and NSE at 4 weeks. From these data, it shows that encapsulated aNSCs differentiated into neurons with different degrees of maturation over this time course. Comparing these data with data from stemness markers, it can be seen that regionality did not significantly impact the expression of neuronal markers as well. However, there was bias toward expression of more mature markers with longer time points as shown in Supplemental Figure S1. Interestingly, these data show that there was a gradual increase in the expression of markers moving from stemness to neuronal lineages in each region of implantation. IHC results (Figure 4B) provide further evidence that aNSCs within the scaffold expressed the neuronal marker, β-III tubulin, in addition to the more mature marker, NeuN. The most striking result to emerge from IHC analysis is the co-expression of the corticospinal tract-specific marker; FezF2 with some cells co-expressing nestin (Figure 4C).

The next sets of IHC analyses focused on the clustered cells within constructs to further understand if there was specific evidence of cross-talk between these cells (Figure 4D). NCAM and synaptophysin, two markers important to cell-to-cell communication and connection, were employed for these analyses. NCAM is an important marker of immature neurons and it is thought to guide cell clusters [55,56] similar to those observed in our scaffolds. Synaptophysin is known to be important in neuronal activities as it ascertains vesicle availability and regulates endocytosis in CNS neurons [57]. The expression of both markers was confirmed by IHC analysis as shown in Figure 4B at 4 weeks and observed in sections throughout the hydrogels within the conduits.

### 3.4. Region-Specific Differentiation of aNSCs

**Figure 5:**
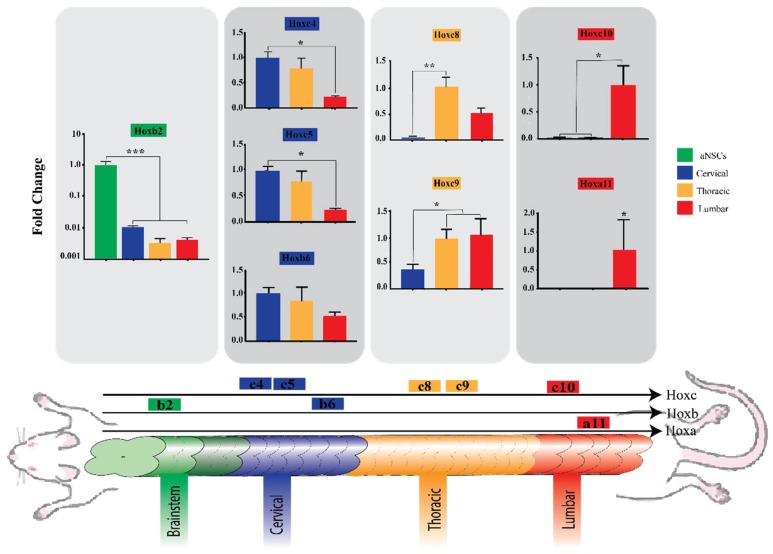
aNSC-encapsulated biomaterial constructs express region-specific markers that correspond to spinal cord regions. Relative quantification analysis using a TaqMan array to compare Hox gene expression in hydrogels harvested from the three implantation regions at 4 weeks, as well as the day 0 aNSCs as a control. Genes are shown in the order of their expression along the craniocaudal axis from left to right. Results show regional trends in the expression of Hox genes. aNSCs of SVZ origin show highest expression of Hoxb2 but failed to express all other presented Hox genes. Cervical implants showed the highest expression of Hoxb3, and Hoxc4. Thoracic implants showed high expression of Hoxc8 and Hoxc9, while lumbar implants showed high expression of Hoxc10, and were the only implants to express Hoxa11. *, **, *** denote significance by one-factor ANOVA (*p* < 0.05, *p* < 0.01, *p* < 0.001). Data are expressed as fold change of the control, based on the region, and analyzed using ∆∆*C*t method. Means ± SEM with *n* = 3.

In the final part of the study we focused on potential for acquisition of region-specific identity by the subcutaneously implanted aNSCs and relied on relative quantification analysis of several Hox genes using commercially available TaqMan arrays. This allowed us to compare Hox gene expression between hydrogels harvested from the three implantation regions at 4 weeks, as well as the starting aNSCs. What can be clearly seen in Figure 5 is a pattern of greater expression of certain Hox genes corresponding to each particular rostro-caudal region. This is evident in the case of cultured aNSCs with no subcutaneous conditioning they expressed the most cranial Hox gene, Hoxb2, and was significantly higher than scaffolds from other regions (*p* = 0.0005), while they failed to express any of the more caudally situated genes. Another example is the caudally suited Hox genes, Hoxc10 and Hoxa11. Hoxc10 expression was significantly higher in scaffolds harvested from lumbar regions than in more rostrally located scaffolds (*p* = 0.0235), while Hoxa11 was expressed exclusively in these lumbar scaffolds. Even though these findings are the most striking observations, the expression of Hox genes in scaffolds harvested from cervical and thoracic regions is interesting as well. In scaffolds from the cervical regions, Hoxc4 was expressed significantly higher than in lumbar implants (*p* = 0.0172). Also, Hoxc5 expression was significantly higher in cervical scaffolds when compared to lumbar scaffolds (*p* = 0.0145). On the other hand, although it was not statistically significant, Hoxb6 expression in cervical scaffolds trended higher than expression in thoracic and lumbar scaffolds (*p* = 0.3475). Moving caudally, Hoxc8 showed higher expression in thoracic implants. The difference between expression in thoracic and cervical scaffolds is statistically significant (*p* = 0.0036), while expression in thoracic scaffolds trended higher than lumbar scaffolds, yet not statistically different (*p* = 0.0802). Moreover, the expression increased further in thoracic and lumbar implants with the more caudal gene, Hoxc9, significantly different from the cervical implants (*p* = 0.0173). Taken together, these results suggest that there is an association between the level of Hox gene expression and scaffold placement, and this association corresponds to the spatial collinearity during morphogenesis.

## 4. Discussion

Despite strong agreement that successful engraftment of NSCs requires preconditioning, there is no agreed upon strategy for preconditioning NSCs before their implantation in a SCI site [25]. Therefore, this study set out with the aim of assessing the influence of subcutaneous environment in combination with our bioscaffold as an instructive strategy for priming aNSCs for future potential application as a SCI treatment. Another objective of the study was to identify whether this approach can also help aNSCs acquire neural identity specific for the region of interest, which would also be of interest for SCI cell-based therapies.

There are agreements between the data expressed by Kadoya et al. [58] and those described here. Through different methods, both motivate for the preconditioning and caudalization of grafts meant to be used for SCI. In this previous study, authors used cells from different sources; rat NSPCs, human NSPCs, and human induced pluripotent stem cells (iPSCs), to graft a SCI site with focus on comparing the caudal versus rostral identity of the graft on regeneration of the corticospinal tract axons. They either used retinoic acid (RA) to induce caudalization of the cells or used cells isolated from spinal cord versus cells from rostral CNS compartments. In each experiment, they compared the caudalized groups to rostralized group, and the three results were similar; the caudalized grafts allowed for corticospinal tract regeneration, while rostralized grafts did not. In current study, we introduce a novel approach that not only induces NSCs caudalization toward spinal cord fate, but also induces them to acquire region-specific identity within spinal cord regions. One of the most interesting findings from our work was that aNSCs encapsulated in a functionalized chitosan-based biomaterial were able to express Hox genes corresponding to the cranio-caudal region of implantation at 4 weeks (Figure 5). This is an important and novel step in stem cell-based therapy because our initial observations suggest that the described approach can guide nascent neurons toward cranio-caudal specific identity. To the best of our knowledge this is the first study that has explored this permissive environment as a potential determinant of implanted aNSC caudalization fate, breaking from the preferred method of in vitro manipulations using RA to direct NSCs or pluripotent-derived NSCs to caudalization fate. Importantly, previous work has shown that the subcutaneous environment has been proven to be clinically safe, sterile and histocompatible [59] and it was associated with favorable outcomes in the short and long term [60,61] for craniotomy graft storage. Subcutaneous maturation of bone grafts has been associated with successful formation of highly mineralized and vascularized bone grafts [62]. A previous report by Kim et al. [63] further supports our findings as it demonstrated that NSCs can change their regional identity marker expression based on local inductive cues. As current knowledge of the developmental cues involved in NSC differentiation is still general, a likely explanation for our observations is that the subcutaneous environment might have retained some of these cues that were originally involved in spinal cord development. It is worth noting that harvested scaffolds at 4 weeks were encapsulated in host tissue, which had not grown past the intact chitosan conduit and was removed before RNA extraction (Figure 2A); however, we cannot completely rule out the influence of remnant subcutaneous tissue on Hox gene data. Nevertheless, a previous study evaluating the expression of Hox gene expression in skin observed that none of the Hox genes they examined were expressed in the adult dermis [64]. Furthermore, Hox gene expression follows different patterns in different organs [37], and the level and order of Hox gene expression that we observed in our results corresponds with the pattern reported by Philippidou and Dasen [65] in developing spinal cords. Importantly, the results of absolute quantification qPCR analysis of 4-week scaffolds did not show significant differences in the expression of neuroepithelial (Figure 3A) or neuronal genes (Figure 4A) which suggests that Hox related region-specific identity is the only response that was impacted by the region of implantation.

Histological analyses revealed another important finding: that aNSCs clustered in cell formations throughout the constructs that highly resemble neural rosettes (Figure 3 and Figure 4), which is a hallmark in CNS development [66,67]. These findings are consistent with and support results that we have reported previously about rosette-like structures seen in similar scaffolds while they were tested for safety and for immune responses after subcutaneous implantation [7,9]. Another study derived neural rosette formations from human and mouse embryonic stem cells (hESCs, mESCs, respectively) with similar morphology and marker expression to the ones we observed in our scaffold [21]. They demonstrated that rosettes can be expanded in vitro without losing their properties and that they can be redirected, using RA, from anterior CNS fates to more caudal fates, including spinal motor neurons, which was marked by increased expression of Hoxb4. Results from this study and from our study demonstrated similar findings, namely that the neural rosettes expressed neuroepithelial markers (PAX6 and Sox1) along with a NSC marker, nestin. Elkabetz and colleagues [21] also proved that rosette stage cells can respond to environmental cues by adopting caudal markers, and expression of these markers was the highest at day 28, while nanog expression gradually decreased and almost disappeared at day 28. This tapering disappearance seen in hESC rosettes may explain the repeatedly undetectable qPCR results observed in our study when measuring nanog gene expression using absolute qPCR in 4-week scaffolds (data not shown). More interesting, Elkabetz and colleagues show that they could induce expression of region-specific markers in the neural rosettes using in vitro patterning cues [21]; however, they were unable to generate spinal cord motor neurons in response to in vitro patterning cues. In our study, corticospinal tract neurons were observed by a FezF2 marker following subcutaneous maturation of 4 weeks (Figure 4C). The presence of more specific neuronal differentiation could be explained by the difference in adult versus embryonic stem cells, or because there are undiscovered patterning cues in the subcutaneous environment that are available to positively impact the differentiation of aNSCs in our bioscaffold. Future work is needed to more deeply characterize the subcutaneous environment, IFN-γ signaling pathways and potential crosstalk that lead to the observed responses.

We developed this approach of subcutaneous maturation of aNSC-seeded biomaterial to control gene expression and neurons’ regional identity acquisition through a bioengineering approach that can replicate and exploit some of the natural process of spinal cord development. The combination of findings provides support for the conceptual premise that subcutaneous implantation of our aNSC-seeded construct can form region-specific neuroepithelium, which differentiate, at least partially, into neurons of different degrees of maturation while also maintaining pools of NSCs/progenitors. Previous reports [7,9,10,52,68] have used similar biomaterials to guide NSC differentiation into different neural progenies. The novelty in our study is subcutaneous maturation and its impact on the differentiation of aNSCs and acquisition of region-specific identity. The results of our approach supports, to some extent, data from a previous study by Kim et al. [63] who studied region-specific markers, in vitro, of NSCs isolated from different CNS compartments and found that NSCs, even though they are specific to CNS compartments, are not committed to one regional fate and they can respond to local environment cues by changing their regional identity. Therefore, endorsing the notion that preconditioning and ectopic maturation of NSCs and progenitors enhance integration into host tissues.

An important translational aspect of our study is the study of the construct degradation time for potential as a graft treatment elsewhere. Operationally, it is crucial to identify a time point when the scaffold can be transferred from the subcutaneous environment where it matures, to a SCI site where it repairs and integrates. The 4-week time point we arrived upon in this study (Figure 2) broadly supports the findings of published work about general consideration and timing of transplantation of NSCs in SCI [44,69]. One study found that implanting NSCs subacutely increased NSCs survival 5-fold [70]. Moreover, 2 to 4 weeks after initial trauma was generally thought to be an optimum window for cell-based therapy [44]. However, these studies primarily were interested in timing after SCI, not necessarily the impact of priming NSCs.

As discussed before, the priming and maturation results, even though they are important, should be interpreted with caution due to the potential influence of the surrounding tissue on the RNA extraction. Another limitation of this study is the presence of chitosan in scaffolds. Chitosan, as a positively-charged polysaccharide, may entrap RNA and decrease the yield. Therefore, the comparison with naïve aNSCs might not have been the most representative. However, the level of expression of studied Hox genes, except for Hoxb2, was significantly higher in scaffolds than in culture aNSCs, suggesting that the material encapsulation most likely did not affect the Hox gene expression pattern observed and the current result are valid as a proof of concept. Moreover, the comparison of expression of multiple Hox genes that represent different regions was done using qPCR as there is no other standard measure to use such as IHC due to lack of availability of antibodies for all Hox genes. Additional future control comparisons should also include NSC-encapsulated hydrogels that are maintained in vitro for similar time points to enable more comprehensive characterization of NSCs behavior.

## 5. Conclusions

This work provides a crucial step toward future research utilizing stem cells for treatments for SCI, as it reports that this novel approach makes predictable region identity acquisition possible and it is more likely to produce better integration when subsequently transplanted. This study also lays the groundwork for further investigation into the role of the subcutaneous environment as an instructive milieu for stem cell differentiation and identity specification. Future work should be undertaken in several directions, but mainly to verify the effectiveness of this strategy in improving the outcome of SCI, as well as to understand the specific signaling pathways and cues that the subcutaneous environment could have retained from organism development and exploit them. The subcutaneous environment is easily accessible and there is abundant room for further progress in both understanding and use of its cues for translational medicine, especially toward grafts for the CNS.

## Supplementary Materials

The following are available online at http://www.mdpi.com/2073-4409/7/10/173/s1.
