# Supplementary material for: Subcutaneous Maturation of Neural Stem Cell-Loaded Hydrogels Forms Region-Specific Neuroepithelium
Source: Cells. 2018 Oct 17;7(10):173. doi: 10.3390/cells7100173 (PMC6210402; doi:10.3390/cells7100173)
Supplement: Supplementary file 1 [file cells-07-00173-s001.pdf]

Supplementary Materials:

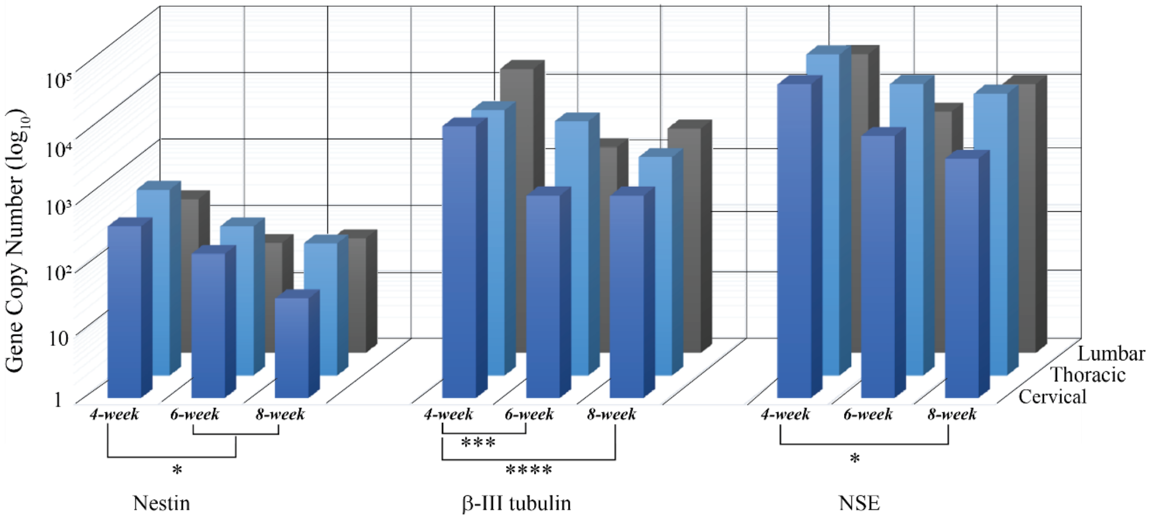

Supplemental Figure 1. aNSCs preferentially mature by 4 weeks as evident by gene expression. qPCR absolute quantification analysis of different genes from three different regions at each timepoint. Nestin expression was significantly different at 4 weeks as compared 6 and 8 weeks (*p* <0.05). -III tubulin expression significantly decreased with longer implantation time as indicated by asterisks (asterisk represents significant difference as follow * *p* <0.05, *** *p* <0.0001, **** *p* <0.00001). To the right, the figure shows significant differences in NSE expression between 4 and 8 weeks (*p* <0.05). Significance by two-way ANOVA, followed by post hoc Tukey HSD test. Mean with *n* =3.

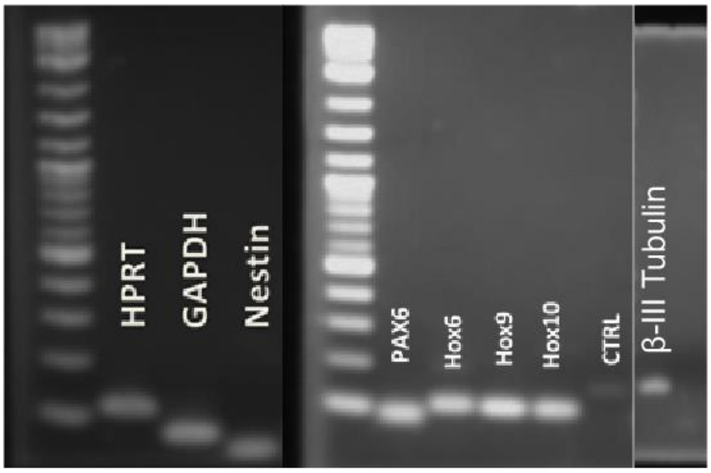

Supplemental Figure 2. Single PCR products were confirmed before cloning. Gel electrophoresis for conventional PCR products confirming that our target genes were amplified as a single product before starting cloning of these product.

Supplemental Table 1. Primer sets for genes used to characterize NSCs differentiation using absolute quantification.

Gene	**Forward Primer (5′ – 3′)**	**Reverse Primer (5′ – 3′)**
GAPDH	AGC TCA TTT CCT GGT ATG ACA A	TAC TCC TTG GAG GCC ATG TA
HPRT	CTC ATG GAC TGA TTA TGG ACA GGA C	GCA GGT CAG CAA AGA ACT TAT AGC C
β-III tubulin	ACT TTA TCT TCG GTC AGA GTC	CTC ACG ACA TCC AGG ACT GA
Nestin	GGC TAC ATA CAG GAC TCT G	AAC CTC GTC CAG GTG TCT G
PAX6	GAA CTT GGA CGG GAA CTG AC	CAA ACA CAC ATG AAC AGT CAG C
NSE	GAG AAC AGC GAA GCT TTG G	AGC CAC ATC CAT ACC AAT CA
Nanog	CAA TGG ATG CTG GGA TAC TCC	TTC TGA ACC TGA GCT ATA AGC AG
